# Preclinical Identification of Poorly Controlled COPD: Patients with a Single Moderate Exacerbation Matter Too

**DOI:** 10.3390/jcm14010022

**Published:** 2024-12-24

**Authors:** José David Maya Viejo, Fernando M. Navarro Ros

**Affiliations:** 1 Centro de Salud de Camas, Santa Maria de Gracia 54, 41900 Camas, Spain; 2 Centro de Salud Malilla, Carrer de Malilla 52D, Quatre Carreres, 46026 Valencia, Spain; fnavarroyros@hotmail.com

**Keywords:** COPD, predictive model, poor control, SABA use, primary care, exacerbations

## Abstract

**Background and Objectives:** Chronic obstructive pulmonary disease (COPD) remains a critical global health challenge, characterized by high morbidity, mortality, and healthcare costs. Current guidelines may overlook patients who present with only one moderate exacerbation or with frequent short-acting beta-agonist (SABA) use. Building on findings from the Seleida study, this research refines the criteria for poor COPD control to include these patients, aiming to improve early identification of high-risk cases in primary care. **Methods:** A retrospectiveand multicenter study is conducted using data from 110 COPD patients in Spain. Poor control is redefined as having at least one moderate exacerbation or as using three or more SABA inhalers annually. Key predictors, such as SABA/short-acting muscarinic antagonist (SAMA) inhalers and antibiotic prescriptions, are identified using logistic regression and LASSO regularization to enhance predictive accuracy. **Results:** The model achieves a good predictive performance, with an AUC-ROC of 0.978, sensitivity of 92.86%, and specificity of 87.50%. Key predictors reliably identify high-risk patients, enabling timely interventions. This study demonstrates a statistically significant association between once-daily inhaler therapies and better COPD control compared to multiple daily doses, supported by chi-square analysis (*p* = 0.008) and binary logistic regression (*p* = 0.018). Nevertheless, the variable ‘daily inhalation frequency’ (1 vs. >1 inhalation/day) was excluded from the final model to prevent overfitting. **Conclusions:** By refining the criteria for COPD control to include patients with at least one moderate exacerbation or frequent SABA use, this model provides a practical tool for early risk stratification in primary care, particularly in resource-limited settings. Early identification of high-risk patients can reduce hospitalizations and healthcare costs, supporting a proactive approach to COPD management. Further validation in larger cohorts is essential to confirm its broader applicability.

## 1. Introduction

Chronic obstructive pulmonary disease (COPD) remains a significant global health challenge, leading to increased morbidity, mortality, and healthcare costs [1,2,3,4]. Characterized by progressive and irreversible airflow limitation, COPD exacerbations(acute episodes of symptom worsening) accelerate disease progression, compromise patient quality of life, and increase healthcare utilization [5,6,7,8]. Thus, preventing exacerbations has become a central goal in COPD management to mitigate poor outcomes, including a heightened mortality risk [9,10].

Over recent decades, treatment strategies have evolved beyond improving lung function to focus on stabilizing the disease and reducing exacerbations [7,11,12]. Early identification of patients at risk of exacerbations, and, consequently, poor disease control, is essential for implementing timely interventions that prevent hospitalizations, reduce clinical and healthcare burdens, and prevent disease progression [9,13]. Current guidelines, such as those from the Global Initiative for Chronic Obstructive Lung Disease (GOLD) and the Spanish COPD guide (GesEPOC), classify patients as high-risk only if they experience two or more moderate exacerbations or a single severe exacerbation [1,11]. However, emerging evidence indicates that these criteria may overlook patients with early signs of instability, such as those who experience only one moderate exacerbation or frequently use short-acting beta-agonists (SABA) [14,15,16,17,18,19,20,21].

Frequent SABA use (≥ 3 canisters annually) is now recognized as a robust indicator of underlying disease instability, correlating with an elevated risk of future exacerbations and increased healthcare utilization [14,21,22]. This reveals a critical gap in current guidelines, which may overlook patients who, despite appearing stable according to conventional criteria, remain at increased risk of rapid disease progression [14,15,16,17,18,19,20,21]. By incorporating SABA use as a marker of early instability, clinicians can optimize decision-making, particularly in primary care settings where advanced diagnostic tools like spirometry are often less accessible [14,21,23,24].

Building on the foundation of the Seleida project, which demonstrated the utility of Electronic Health Records (EHRs) in predicting poor disease control in patients suffering from COPD and asthma [25], this study refines the concept of poor control by including patients presenting with a single moderate exacerbation or moderate-to-high SABA use. By leveraging routinely available clinical data, such as rescue medication use and antibiotic prescriptions, this updated model aims to enhance the early identification of high-risk COPD patients, especially in resource-limited primary care settings.

This refined approach not only aligns with the need for proactive patient management, but also offers a practical strategy to reduce exacerbations, hospitalizations, and healthcare costs. The following sections outline the methodology used to refine the predictive model, validate its performance, and assess its potential for improving COPD management in clinical practice.

## 2. Materials and Methods

### 2.1. Study Design and Data Source

This retrospective and multicenter study utilized anonymized EHRs from the Spanish *Sistema Nacional de Salud* (SNS) to develop a predictive model to identify poorly controlled COPD patients based on readily accessible clinical variables [25]. Data were randomly sampled from two primary care centers to ensure a representative cohort of real-world COPD cases. Building on the original Seleida study—a prior observational, non-interventional multicenter analysis—this work refined the definition of poor disease control by including patients with a single moderate exacerbation or moderate-to-high SABA use, addressing gaps in the traditional criteria [14,15,16,17,18,19,20,21].

This analysis was derived from the original Seleida study, which obtained prior approval from the ethics committees of both participating centers. All data were anonymized in compliance with data protection regulations to ensure confidentiality.

### 2.2. Patient Selection and Variables Collected

Eligible patients were aged 40 years to 80 years, with a confirmed diagnosis of COPD or documented COPD treatment for at least three months per year over the past two years. Exclusion criteria included active malignancies, patients in palliative care, those with asthma–COPD overlap syndrome (ACOS), chronic users of systemic corticosteroids, recipients of biologic therapies, and participants in clinical trials. Additionally, bedridden or severely disabled patients were also excluded to minimize the number of confounding factors affecting disease control. Only patients with complete, up-to-date clinical records and consistent follow-ups during the study period were included to ensure robust predictive analyses.

Key clinical variables extracted from EHRs included demographic data (age, sex, and province of residence), anthropometric measurements (height, weight, body mass index [BMI]), and relevant comorbidities (e.g., smoking status, sleep apnea, obesity, cardiovascular diseases). COPD-specific variables encompassed daily inhalation frequency, use of rescue medications (annual SABA and short-acting muscarinic antagonist [SAMA] prescriptions), the number of exacerbations in the past year (moderate or severe), as well as the number of emergency department visits or physician consultations for respiratory issues. Data on systemic corticosteroid use (converted to prednisone-equivalent doses) and antibiotic prescriptions for bronchitis or exacerbations were also collected, along with blood eosinophil counts. A comprehensive description of the variables collected in the Seleida study has been previously published [25].

All patient medications included in this study were evaluated using annual dispensing data recorded by pharmacies and integrated into the patients’ EHRs. This method provides an accurate measure of actual medication use, minimizing biases associated with non-adherence or unrecorded prescriptions.

### 2.3. Model Definition

Current COPD risk and control models, such as those proposed in the GOLD 2024 and GesEPOC 2021 frameworks, primarily rely on spirometry, symptom assessments, and exacerbation history [1,11]. However, these approaches may fail to identify patients at risk who experience moderate exacerbations or exhibit elevated SABA use, both of which are strongly linked to worsening clinical outcomes [14,15,16,17,18,19,20,21]. Moderate exacerbations—defined as episodes requiring systemic corticosteroids and/or antibiotics without hospitalization—and the use of three or more SABA canisters annually are established predictors of increased exacerbation risk and suboptimal disease management [9,13,14,15,16,17,18,19,20,21].

The core premise of our model is that a well-controlled COPD patient should not require frequent rescue medications or have experienced exacerbations within the past year. To address existing gaps, we developed a predictive model that redefines COPD control by incorporating moderate exacerbations and frequent SABA use as primary indicators. This model identifies patients with a single moderate exacerbation, one severe exacerbation, or frequent SABA use (≥ 3 canisters annually), providing a more precise tool for detecting patients who may be overlooked by traditional criteria.

In our model, control is defined by the probability of future exacerbations, with higher probabilities indicating a poorer control. To enhance its practicality in primary care settings, we intentionally excluded spirometry data, focusing instead on easily accessible clinical markers. This decision broadens its applicability, especially in settings where spirometry is underutilized or unavailable [23]. The refined approach aims to support proactive management, reduce exacerbations, slow disease progression, and alleviate healthcare costs.

By integrating previously overlooked variables, the model enhances early identification of high-risk patients, enabling timely interventions. Its focus on moderate exacerbations and frequent SABA use aligns with current evidence, underscoring their predictive value for adverse outcomes [14,16,26]. This adaptability to real-world primary care settings increases the model’s potential to improve COPD management through early intervention, ultimately enhancing patient outcomes and reducing the overall healthcare burden [27].

### 2.4. Statistical Analysis

After excluding four patients from the initial cohort of 110 to avoid bias (see Section 2.6), all statistical analyses were conducted using the R software (version 4.3.4.2) [28]. This platform was chosen for its robust capabilities to handle complex predictive models essential for identifying high-risk COPD patients [28]. A significance level of 0.05 was chosen, and variables with more than 50% missing data were excluded to ensure model stability [29]. The analysis was divided into two phases to enhance the predictive accuracy.

#### 2.4.1. Phase 1

Associations between clinical variables and disease control were assessed using chi-square and Fisher’s exact tests for categorical variables and Wilcoxon signed-rank tests for continuous variables [30]. To control for multiple comparisons and reduce the risk of Type I errors, the Benjamini–Hochberg correction was applied [27]. This phase aimed to identify clinically relevant variables, ensuring that only the most significant predictors were carried forward to the subsequent model for targeted interventions.

#### 2.4.2. Phase 2

Variables identified as significant in Phase 1 (adjusted *p*-value < 0.05) were included in a logistic regression model using the *glmnet* package [31]. To prevent overfitting, LASSO (least absolute shrinkage and selection operator) regularization was applied, optimizing model performance by selecting relevant variables while shrinking coefficients [31]. LASSO was preferred over the Ridge regression, which only shrinks coefficients, as it also performs variable selection, resulting in a more parsimonious and interpretable model. Although Elastic Net combines aspects of both methods, its added complexity was deemed unnecessary given our dataset [31].

The model was further refined using a backward stepwise approach guided by the Akaike information criterion (AIC) [27]. Predictive performance was evaluated using sensitivity, specificity, positive predictive value (PPV), negative predictive value (NPV), and accuracy, employing an 80/20 training–validation split [32]. Bootstrapping with 1000 iterations was applied to confirm the stability of the coefficients.

### 2.5. Definition of Minimal Sample Size for Model Validation

Sample size calculations were grounded on data from the AVOIDEX study, which reported that 48.2% of the patients were non-exacerbators [33]. This approach ensured that the study had sufficient power to detect clinically relevant predictors.

#### 2.5.1. Determination of Minimum Events in the Training Set

Based on the “10 events per predictor variable” rule, the training set (80% of the dataset) required at least 20 events to ensure stable estimates [27]. With two predictor variables, this threshold was comfortably met, allowing for the reliable identification of patients at risk.

#### 2.5.2. Calculation of Total Sample Size

To validate the model’s performance, we applied the formula 0.2 × *n* × 0.482 ≥ 10, which yielded a required sample size of at least 104 patients [27]. This calculation was essential to ensure that the model’s findings could be generalized to clinical practice, where timely identification of high-risk patients is crucial for early intervention.

#### 2.5.3. Verification of Events in the Training Set

The training set contained approximately 40 events (0.8 × 104 × 0.482 ≈ 40), exceeding the minimum requirement of 20 events, thereby confirming model stability. The total sample size of 106 patients provided robust internal validity. Although the McNemar’s test indicated no significant differences in error rates compared to reference classifiers, suggesting strong predictive reliability, further studies with larger cohorts could enhance the model’s precision [34,35].

### 2.6. Limitations

This model focuses on accessible clinical variables, but several limitations must be acknowledged. The exclusion of spirometry data, while enhancing practicality in primary care, may reduce precision in complex cases where lung function assessments are crucial [36,37]. Although spirometry remains the gold standard for COPD diagnosis, its underutilization in primary care is often due to resource and time constraints [24,38]. This underscores the need for pragmatic tools that can function effectively without spirometry [37].

The retrospective design may introduce biases, such as incomplete or inconsistent data. To overcome this, we employed a widely accepted approach in clinical and epidemiological research in which variables with more than 50% missing data were excluded [29]. This strategy ensures model stability, minimizes the risk of introducing artificial patterns, and enhances the robustness of the results. By focusing on variables with sufficient information, the analysis meets the principles of predictive modeling, prioritizing interpretability and reliability when working with real-world data. Furthermore, imputation techniques, such as multiple imputation, were not applied due to the retrospective design and the associated risk of changing underlying associations. Additionally, four patients without documented COPD treatment in the past year were removed to prevent confounding effects from potential data entry errors, overdiagnosis, untreated mild cases, poor adherence, unrecorded private sector care, or failure to deregister deceased patients.

While the model relies on rescue medication use and exacerbation history to guide interventions, further validation in diverse populations is essential to confirm its generalizability. Expanding the cohorts to include a broader range of demographics would enhance its applicability. Future iterations could incorporate spirometry data or patient-reported outcomes to increase precision, especially in more complex cases [36].

Despite these limitations, the model effectively identifies key predictors of poor COPD control using readily available data, addressing gaps in current diagnostic criteria [25]. This highlights its potential for early detection and proactive management in the context of primary care, with a focus on prioritizing interventions for poorly controlled patients.

## 3. Results

Our findings underscore the critical need for early, proactive interventions in COPD management, particularly following a patient’s first exacerbation. These episodes not only accelerate disease progression, but also increase healthcare utilization and reduce quality of life [11,20]. Early identification of poorly controlled patients is essential, as they face a significantly higher risk of rapid decline, imposing a substantial burden on healthcare systems [26].

The study demonstrates that promptly identifying patients after even a single moderate exacerbation allows for timely interventions to prevent further deterioration [20,21]. By integrating the exacerbation history with key biomarkers, such as eosinophil levels, we enhanced the precision of personalized treatment strategies [36]. This approach aligns with evidence supporting individualized interventions, optimizing patient outcomes and reducing healthcare costs.

The following results highlight the need for a predictive model to prioritize patients with poor control, enabling targeted interventions that can mitigate disease progression and alleviate the strain on healthcare resources.

### 3.1. Patient Characteristics

From the initial cohort of 110 patients, four from the Valencia cohort were excluded due to a lack of prescribed maintenance or rescue medication in the previous year. These patients, initially classified as having good COPD control, were excluded to prevent potential bias, as their apparent control status likely reflected incomplete treatment records rather than their actual clinical condition (see Section 2.6) [29]. This adjustment resulted in a final cohort of 106 patients, 60 from Seville and the remainder from Valencia, with a mean age of 68.8 ± 8.2 years, predominantly male (72.6%).

**Figure 1:**
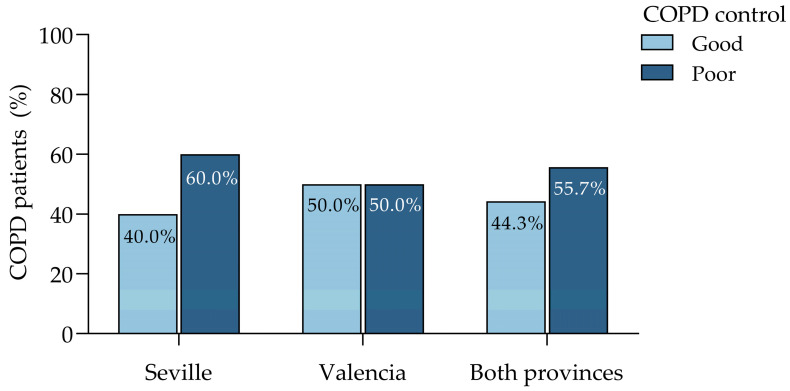
COPD control. COPD, chronic obstructive pulmonary disease.

Using our refined criteria for poor COPD control—defined as at least one moderate exacerbation, one severe exacerbation requiring hospitalization, or an annual use of three or more SABA canisters—55.7% of the patients were classified as poorly controlled, while 44.3% were well-controlled (Figure 1). These rates slightly differ from those reported in the AVOIDEX study (51.8% poorly controlled, 48.2% well-controlled) [33], supporting the external validity of our findings. The higher proportion of poorly controlled patients in our cohort ensured a sufficient number of events to validate the model, meeting the “10 events per predictor variable” threshold [27].

Patients were distributed across two healthcare centers in Seville and Valencia. The Seville center had a higher proportion of poorly controlled patients (60.0%) compared to Valencia (50.0%), while Valencia had a greater proportion of well-controlled cases (50.0% vs. 40.0% in Seville) (Figure 1). However, this difference was not statistically significant (χ² = 1.055, *p* = 0.304), suggesting minimal regional variations.

Aside from the control criteria, baseline characteristics were largely similar between the two groups. These findings underscore the need for more targeted strategies to identify patients at risk of poor control, particularly in regions like Seville, where a higher burden of poorly controlled cases was observed.

### 3.2. COPD Control: Definition by SABA Use and Exacerbation Frequency

**Figure 2:**
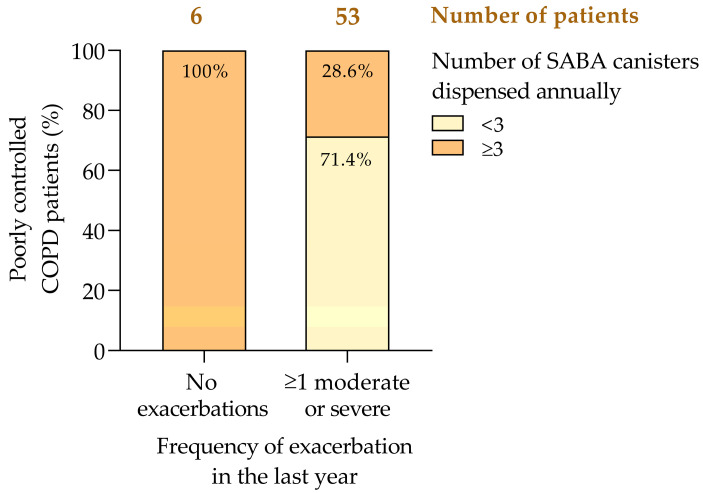
Contribution of SABA use and exacerbation frequency in the study sample. COPD, chronic obstructive pulmonary disease; SABA, short-acting beta-agonist.

Of the 59 patients classified as having a poorly controlled disease, six (10.2%) were identified exclusively by moderate-to-high SABA use (≥3 canisters annually), despite the absence of exacerbations in the last year (Figure 2). In contrast, 38 patients (64.4%) were classified based only on the presence of ≥1 moderate exacerbation with a consumption of SABA canisters < 3/year [71.4% of the “at least one moderate exacerbation” (Figure 2)]. Finally, 15 patients (25.4%) met both criteria, a high SABA use and at least one moderate exacerbation [28.6% of the “≥1 moderate exacerbation” and “≥3 SABA canisters annually” (Figure 2)].

These findings reveal a clear heterogeneity in the profiles of poorly controlled COPD patients. The subgroup identified by SABA use alone featured the role of rescue medication dependence as an isolated marker of poor control, while the larger proportion driven only by exacerbations underscored the persistence of the symptoms despite a low SABA use. The overlap between the criteria, observed in a quarter of the patients, reflected a more severe impairment of disease control and pointed out the importance of a multifactorial approach. Integrating both variables will provide a more comprehensive framework for evaluating COPD control, facilitating better patient stratification and targeted interventions in clinical practice.

### 3.3. Inhalation Therapy Regimens and COPD Control

**Figure 3:**
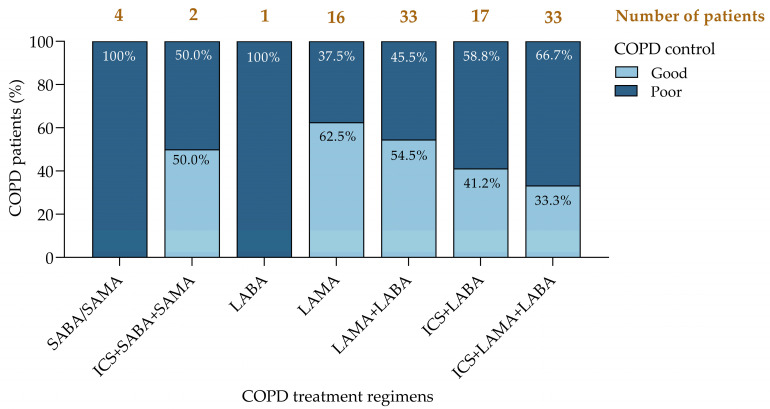
COPD control by treatment regimen. COPD, chronic obstructive pulmonary disease; ICS, inhaled corticosteroids; LABA, long-acting beta-agonists; LAMA, long-acting muscarinic antagonists; SABA, short-acting beta-agonists.

The treatment regimen of COPD patients showed a tendency related with the degree of disease control (Figure 3). Long-acting beta-agonist (LABA), dual bronchodilation (LABA plus long-acting muscarinic antagonist [LAMA]), and inhaled corticosteroid (ICS) regimens showed the highest association with poor disease control probably reflecting a subgroup of patients with more severe diseases or more frequent exacerbations (Figure 3). Similarly, SABA or SAMA in monotherapy was associated with 100% poor control, highlighting its inadequacy for achieving proper disease management. In contrast, the LAMA treatment as a monotherapy showed the lowest proportion of poor control (37.5%), suggesting its effectiveness in patients with less severe diseases or with specific phenotypic profiles (Figure 3).

Our findings must be interpreted with caution, since treatment selection depends on the patient’s phenotype and clinical profile, including disease severity, exacerbation frequency, and inflammatory biomarkers. Consequently, comparisons between treatment groups are limited unless patients share similar phenotypic characteristics. Stratifying patients by phenotype in future studies will allow a more accurate evaluation of treatment effectiveness and guide targeted therapeutic strategies. Moreover, the statistical analysis (Pearson’s chi-square, *p* = 0.161) indicates that the observed differences were not significant, suggesting random variation. The Spearman correlation coefficient (r = 0.121; *p* = 0.215) supports this weak association.

### 3.4. COPD Treatment Strategies: The Relevance of Daily Dosage

**Figure 4:**
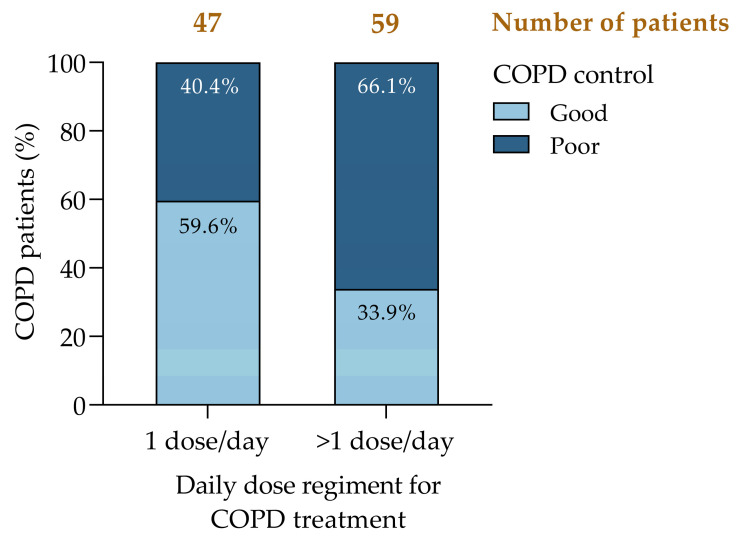
COPD control by daily dose regimen in basal treatment. COPD, chronic obstructive pulmonary disease.

We found a significant association between once-daily inhaler therapy regimens and improved COPD control (Figure 4). Patients on once-daily regimes exhibited a higher proportion of good disease control (59.6%) compared to those requiring multiple daily doses (33.9%) (Figure 4). In contrast, poor COPD control was significantly more frequent among patients requiring multiple inhalations per day (66.1%). The relevance of our findings was confirmed by a statistical analysis: a chi-squared test yielded a value of 6.96 (*p* = 0.008), allowing rejection of the null hypothesis of independence, and the Cramer’s V coefficient was 0.256, highlighting a moderate association and emphasizing the clinical relevance of dosing simplification.

To explore whether treatment adherence was influenced by the dosing frequency, we evaluated the proportion of days covered (PDC) for treatment. A Mann–Whitney U test revealed no significant differences between groups (U = 1371.5, Z = −0.132, *p* = 0.895). Moreover, the nearly identical mean ranks (53.07 for once-daily regimens and 53.85 for multiple doses) confirm that adherence was similar despite the dosing regimen.

The results suggest that simplifying COPD treatment to a once-daily regimen, when clinically appropriate, may reduce the therapeutic burden and improve disease control. Further studies with larger and more diverse cohorts are necessary to validate these findings and explore the mechanisms underlying this association.

### 3.5. Exacerbations and Healthcare Utilization

The frequency of exacerbations was strongly associated with an increased healthcare utilization, consistent with previous studies [8,39]. A one-tailed paired *t*-test confirmed a significant correlation between annual exacerbations and respiratory consultations (*p* = 0.010), indicating that patients with frequent exacerbations required more healthcare resources.

**Figure 5:**
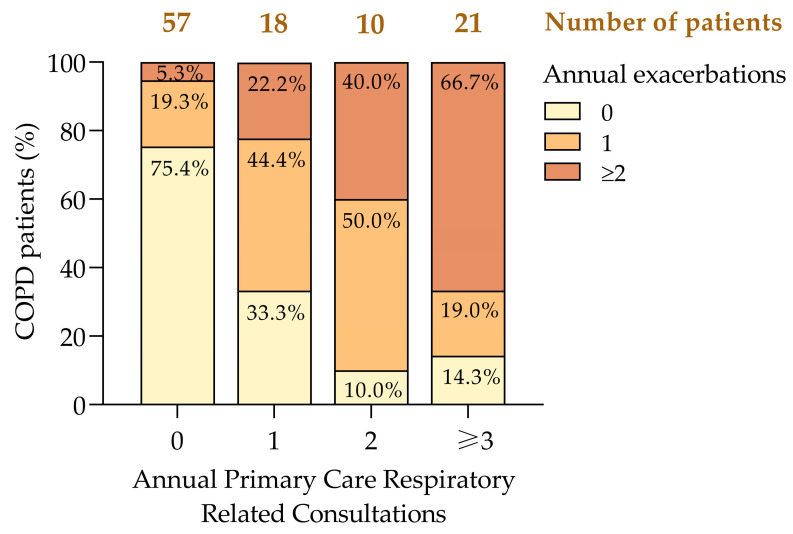
Annual primary care consultations based on the number of COPD exacerbations in the previous year. COPD, chronic obstructive pulmonary disease.

Our analysis revealed a clear trend: as the frequency of the consultations increased, so did the proportion of patients experiencing multiple exacerbations (Figure 5). Notably, 66.7% of the patients with three or more consultations had ≥2 exacerbations compared to only 5.3% among those who required no consultations (Figure 5). Conversely, 75.4% of the patients who did not require consultations experienced no exacerbations, highlighting the link between effective COPD control and reduced healthcare demands.

The data also showed a progressive increase in the proportion of patients with ≥ 2 exacerbations as consultation frequency rose, from 5.3% among those requiring no consultations to 66.7% among those requiring three or more consultations. Additionally, 44.4% of the patients with one consultation and 50.0% of those with two consultations experienced a single exacerbation, suggesting that early intervention in these groups could prevent further disease progression.

A moderate-to-strong positive correlation (r = 0.615, *p* < 0.001) confirmed that patients with more exacerbations had greater healthcare demands. These findings align with prior research demonstrating a high prevalence of exacerbations among poorly controlled patients [20,26,40], underscoring the need for targeted interventions to reduce both exacerbations and the associated healthcare burden.

### 3.6. Medication Data on COPD Exacerbations

In poorly controlled COPD, 84.8% of the patients with at least one moderate exacerbation received antibiotics, compared to none among those without exacerbations. While this demonstrates a clear association between exacerbations and antibiotic use, the absence of microbiological data limits interpretation and raises concerns about potential overtreatment. Additionally, prospective studies incorporating microbiological analyses are necessary to ensure appropriate prescription practices.

**Figure 6:**
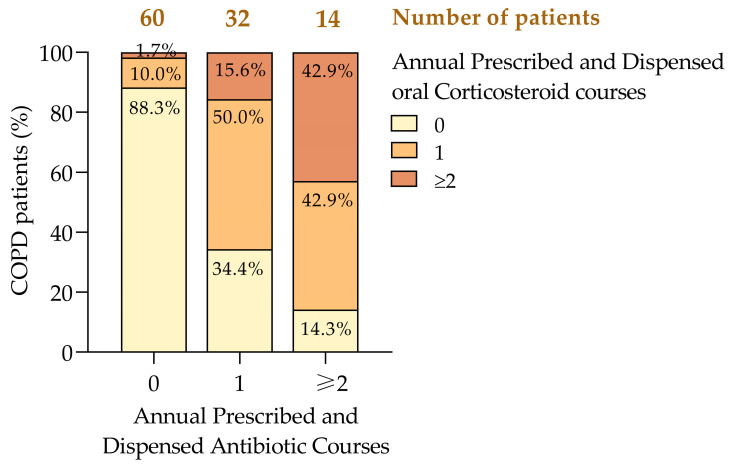
Cross-tabulation of annual prescriptions of antibiotic and oral corticosteroid courses in COPD patients. COPD, chronic obstructive pulmonary disease.

COPD exacerbations were primarily managed with antibiotics and oral corticosteroids [1,11]. Among the patients who did not require antibiotics, 88.3% also did not need corticosteroids, while 10% required one course and 1.7% needed two or more courses of corticosteroids. In contrast, among those who had been prescribed a single antibiotic course, 50% also required one corticosteroid course, and 15.6% needed two or more (Figure 6). Patients requiring two or more antibiotic courses were more likely to need corticosteroids, with 42.9% requiring one course and another 42.9% requiring two or more. This pattern suggests that frequent antibiotic use correlates with more severe exacerbations that also necessitate corticosteroid treatment [6,8].

Although a paired *t*-test did not reveal a significant difference between antibiotic and corticosteroid use (*p* = 0.207), a Pearson’s correlation coefficient (r = 0.623, *p* < 0.001) indicated a moderate-to-strong positive association, suggesting that higher antibiotic use is associated with an increased need for corticosteroids. The bivariate linear regression further supported this, showing that each additional antibiotic course was associated with a 0.603 unit increase in corticosteroid use (*p* < 0.001).

Effect sizes (Cohen’s d = 0.613, Hedges’ g = 0.617) demonstrated that patients with poorly controlled COPD were significantly more likely to require multiple courses of antibiotics and corticosteroids, along with an increased SABA use [14,15,41]. These findings underscore the need for personalized management strategies to reduce exacerbations and optimize treatment, supporting evidence for tailored interventions aimed at improving patient outcomes and reducing healthcare costs [26,36].

### 3.7. Eosinophil Levels and Disease Phenotypes

Eosinophil counts were available for 87.7% of the patients, with a mean of 230.9 ± 137.8 cells/µL. The majority (59.4%) had levels between 100 cells/µL and 300 cells/µL, while 22.6% had counts ≥ 300 cells/µL. According to both GOLD and GesEPOC guidelines [1,11], eosinophil levels ≥ 300 cells/µL strongly support the use of inhaled corticosteroids (ICSs) due to the associated inflammatory burden. For patients with levels between 100 cells/µL and 300 cells/µL, ICSs may also be beneficial, particularly for those who have experienced at least one moderate exacerbation in the past year [1,14,20,42].

**Figure 7:**
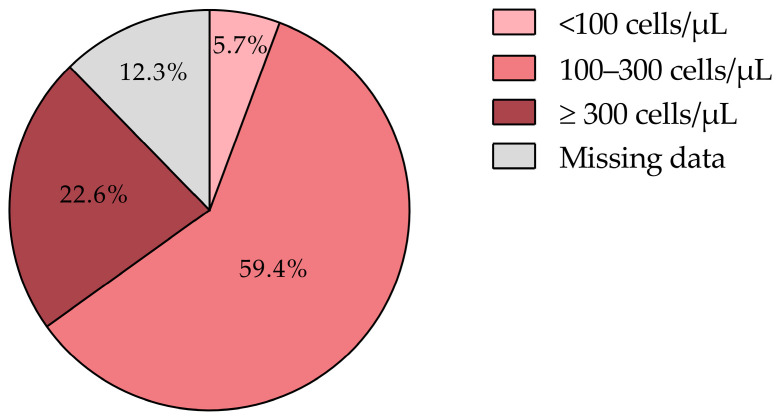
Average annual eosinophil levels in COPD patients by value ranges. COPD, chronic obstructive pulmonary disease.

In alignment with the GOLD 2024 guidelines, ICSs are recommended for patients with a history of hospitalization due to COPD exacerbations, two or more moderate exacerbations annually, or eosinophil counts ≥ 300 cells/µL. Conversely, ICSs should be avoided in patients with counts < 100 cells/µL or with a history of mycobacterial infections or recurrent pneumonia [43]. In our cohort, only 5.7% had eosinophil counts < 100 cells/µL (Figure 7), suggesting that ICS therapy may not be necessary for this subgroup.

Overall, 82.0% of the patients showed eosinophil counts of ≥100 cells/μL, a key marker for identifying candidates who may benefit from ICS therapy when certain criteria are met. According to the GOLD 2024 guidelines, patients with eosinophil counts ≥ 100 cells/μL who have been previously treated with long-acting bronchodilators and who have experienced at least one moderate exacerbation in the prior year are likely to experience improved outcomes with the addition of ICSs. This underscores the combined role of eosinophil levels and exacerbation history in guiding individualized COPD management. By integrating these parameters into treatment decisions, clinicians can implement more precise interventions aimed at reducing exacerbations and improving patient outcomes [14,20,42,44]. However, the absence of eosinophil data for 12.3% of the patients represents a limitation, highlighting the importance of consistent monitoring to optimize therapeutic strategies.

### 3.8. COPD Phenotypes Based on Exacerbation Frequency and Annual SABA Use

**Figure 8:**
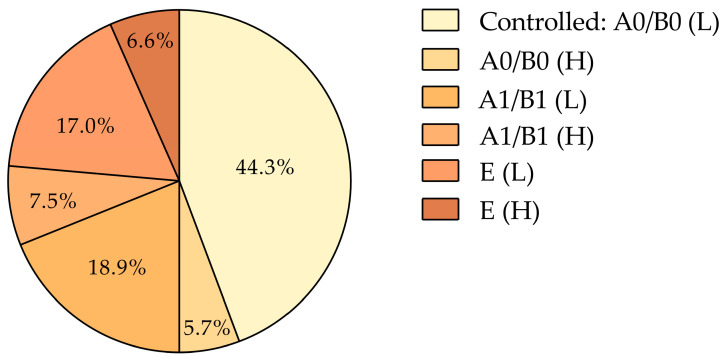
COPD phenotypes according to the number of exacerbations and annual consumption of SABA. COPD, chronic obstructive pulmonary disease; SABA, short-acting β-agonists.

Patients were classified into six distinct phenotypic subgroups according to the GOLD 2024 ABE classification [1] and their annual SABA consumption (Figure 8):

- Controlled (A0/B0 [L]): no exacerbations and a low SABA use (44.3%).
- A0/B0 (H): no exacerbations but a high SABA use (5.7%).
- A1/B1 (L): one moderate exacerbation with a low SABA use (18.9%).
- A1/B1 (H): one moderate exacerbation with a high SABA use (7.5%).
- E (L): two or more exacerbations with a low SABA use (17.0%).
- E (H): two or more exacerbations with a high SABA use (6.6%).

This classification system stratifies patients based on exacerbation frequency and SABA consumption, where the first letter indicates the number of exacerbations and the letter in brackets denotes the SABA use (‘L’ for low, <3 canisters/year; ‘H’ for high, ≥3 canisters/year). An elevated SABA use, particularly among patients suffering from frequent exacerbations, correlates with a poor disease control and suggests a need for more intensive management [14,15,21].

Notably, a high SABA use in patients without exacerbations (A0/B0 [H]) likely reflects an increased symptom burden [14,36], indicating that these patients may benefit from a reevaluation of their treatment regimen. Unfortunately, this study does not allow for a distinction between group A and group B due to the absence of COPD assessment test (CAT) or modified medical research council scale (mMRC) assessments. This stratification aligns with the GOLD ABE framework, providing a structured approach to tailor interventions based on individual patient risk profiles. By integrating both the exacerbation history and the SABA use, this classification system supports a personalized approach to COPD management, with the goal of optimizing outcomes and reducing exacerbation rates [15,20].

### 3.9. Determination of Clinical Variables for Predicting Poor Control of COPD Disease

We developed a predictive model for poor COPD control using robust statistical techniques to ensure stability and accuracy [35,38]. Four untreated patients with no recorded daily inhalations were excluded to prevent potential confounding effects from overdiagnosis, mild disease, or incomplete records [31]. This exclusion ensured that the model focused solely on actively treated patients, thereby enhancing data reliability.

#### 3.9.1. Statistical Analysis and Model Development

Univariate analyses identified significant associations between poor control and several variables, including SABA/SAMA use (*p* < 0.001), respiratory consultations (*p* = 0.010), annual corticosteroid courses (*p* < 0.001), prednisone doses (*p* = 0.004), and antibiotic courses (*p* < 0.001). Age showed a weaker association (*p* = 0.049). To ensure robustness, we applied the Benjamini–Hochberg correction and addressed multicollinearity using the variance inflation factor (VIF) [27].

**Figure 9:**
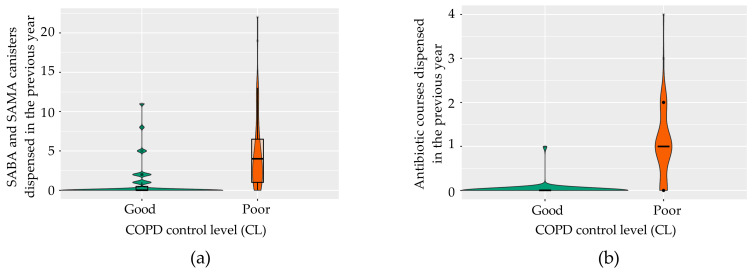
Violin plot of the key predictive variables in the final COPD control model. (**a**) Number of SABA and SAMA canisters dispensed in the last year according to the status of disease control; (**b**) number of antibiotic courses dispensed in patients with good and poor disease control. COPD, chronic obstructive pulmonary disease; SABA, short-acting β-agonists; SAMA, short-acting muscarinic antagonists.

Subsequently, we developed a binary logistic regression model using the Akaike information criterion (AIC) for variable selection [31]. The analysis identified two primary predictors: the annual count of SABA/SAMA inhalers dispensed, and the number of antibiotic courses prescribed for exacerbations (Figure 9 and Table 1). The model demonstrated strong reliability, with a residual deviance of 44.33 and an AIC score of 50.33.

Although the transformed variable ‘daily inhalation frequency’ (categorized as 1 vs. >1 inhalation/day) demonstrated, through binary logistic regression, that patients requiring only one inhalation per day had significantly better control than those requiring multiple doses (*p* = 0.018), it was excluded to prevent overfitting due to high bias and increased standard error.

By focusing on key variables—specifically, rescue medication use and antibiotic prescriptions—the model remains both clinically relevant and easy to interpret for primary care providers. Utilizing data readily available from EHRs enables clinicians to promptly identify at-risk patients, facilitating timely, data-driven interventions without the need for complex diagnostics.

#### 3.9.2. Key Findings and Model Optimization

Both predictors(SABA/SAMA inhaler use and antibiotic courses) were statistically significant and clinically relevant markers of disease instability (Table 1) [44,45]. To enhance the model, we applied the LASSO regularization, which improved interpretability by selecting the most relevant variables [31]. Bootstrap resampling (1000 iterations) confirmed the stability of the model’s coefficients (Table 2) [27]. The positive coefficients for both predictors indicate that an increased medication use correlates with a higher likelihood of poor control, underscoring the need for targeted management strategies.

#### 3.9.3. Model Performance

The final model achieved an accuracy of 90.91% [95% CI: 70.84–98.88], with a sensitivity of 92.86%, a specificity of 87.50%, a PPV of 92.86%, and an NPV of 87.50%. The post-LASSO kappa coefficient of 80.36% confirmed a strong predictive reliability. The model demonstrated a substantial discriminative ability, with an AUC-ROC of 0.978. Additionally, a high positive likelihood ratio (LR+ of 7.43) indicated a strong predictive power for identifying poor control, while a low negative likelihood ratio (LR- of 0.082) minimized the risk of misclassifying patients with good control.

#### 3.9.4. Predictive Equation

Following the LASSO regularization, the final predictive equation became:

$$y=\frac{1}{1+e^{-f(x)}}$$

$$f\left(x\right)=-1.873+0.427\cdot \left[SABA+SAMA\right]+3.654\cdot \left[Antibiotic\_courses\right].$$

[*SABA + SAMA*]: total number of SABA and SAMA canisters prescribed and dispensed in the previous year.

[*Antibiotic_courses*]: number of antibiotic courses prescribed and dispensed in the previous year due to bronchitis or COPD exacerbations.

The model classifies patients based on their predicted probability (y) of poor COPD control, using a threshold of 0.50. Patients with probabilities above this threshold are classified as poorly controlled, while those below are deemed to be well-controlled. Further adjustments to the cutoff point using the ROC curve analysis and the Youden’s J index did not result in significant improvements, confirming the model’s optimal performance.

## 4. Discussion

This study introduces a clinically practical model for identifying poorly controlled COPD patients in primary care settings, demonstrating exceptional predictive performance with an AUC-ROC of 0.978, along with a high sensitivity (92.86%) and specificity (87.50%). By focusing on straightforward yet robust markers, such as SABA/SAMA use and antibiotic dispensations [25], the model provides a powerful tool for early detection, particularly in settings where access to advanced diagnostic markers is limited [46].

A key insight from our analysis reveals that many patients, despite appearing stable under the traditional criteria, may exhibit early signs of poor control when assessed according to their medication use patterns. For instance, a COPD patient who has experienced only one moderate exacerbation in the past year but frequent SABA use (≥ 3 canisters annually) would typically be classified as stable according to the current guidelines. However, our model would identify this patient as high-risk, prompting clinicians to optimize maintenance therapy or initiate preventive interventions. This proactive approach could significantly reduce future exacerbations and hospitalizations, bridging critical gaps in conventional diagnostic practices while enabling more personalized management strategies.

Our findings underscore that patients on a maintenance regimen of a single daily inhalation exhibit significantly better disease control compared to those requiring multiple inhalations, with a statistically significant association demonstrated by a chi-squared analysis (*p* = 0.008) and further confirmed by binary logistic regression (*p* = 0.018) [19]. This observation suggests that streamlined treatment strategies align with the shift toward personalized, proactive COPD management and may enhance patient adherence. However, in order to prevent overfitting or loss of consistency, we decided to exclude this variable from the predictive model because it remains uncertain whether the superior control observed in these patients was due to an inherently lower symptomatic burden or whether the regimen’s simplicity itself was responsible for the better outcomes [1,21,47,48]. Further research is needed to elucidate the relationship between inhalation frequency and clinical stability.

Additionally, the model’s reliance on medication patterns, particularly rescue medication use and annual antibiotic prescriptions, extends beyond prediction by offering a pathway for preclinical phenotyping. This approach could facilitate the early identification of high-risk patients, enabling targeted interventions tailored to specific COPD phenotypes [49,50]. By leveraging readily available EHRs, the model can be seamlessly integrated into various healthcare settings, enhancing its practicality and scalability [25].

Implementing this model could yield substantial cost savings by reducing the frequency of exacerbations, hospitalizations, and emergency department visits [51]. Early identification of high-risk patients enables a more efficient allocation of resources, which is crucial for sustainable COPD management, particularly in resource-constrained settings [52]. For example, preventing a single hospitalization can result in significant cost savings, thereby alleviating the pressure on healthcare systems.

While excluding spirometry data enhances the model’s feasibility in primary care settings, it may limit precision in complex cases [53]. Future iterations could incorporate real-time patient data and leverage machine learning algorithms to refine predictions, enabling continuous monitoring and adaptive interventions [36,54,55,56]. Moreover, integrating patient-reported outcomes could provide a more comprehensive assessment of disease control, further enhancing the model’s utility.

To confirm its generalizability, expanding the validation cohort to include more diverse populations is essential. A broader implementation across different healthcare contexts will help establish its utility, promoting a more personalized approach to COPD management. As healthcare systems face increasing pressures, this model offers a scalable solution to shift from reactive to proactive care, ultimately optimizing patient outcomes, reducing costs, and ensuring a more sustainable approach to COPD management.

## 5. Conclusions

This study introduces a practical predictive model for early identification of poorly controlled COPD patients in primary care settings. By focusing on simple yet robust markers—specifically, SABA/SAMA use and antibiotic prescriptions—the model achieves a high sensitivity (92.86%) and specificity (87.50%), allowing for proactive interventions that reduce disease progression and alleviate healthcare burdens [17,25,30].

By expanding the criteria to include patients who have experienced a single moderate exacerbation or who rely on frequent SABA use, the model addresses gaps in current diagnostic approaches and aligns with the shift toward personalized medicine [14,15,16,17,18,19,20,21]. It leverages routinely collected EHR data, facilitating its seamless integration into diverse healthcare settings and supporting evidence-based decision-making.

Further validation in larger, diverse populations is needed to confirm its generalizability potential. Future iterations could incorporate machine learning and real-time data to enhance the predictive capability and enable continuous patient monitoring [36,56]. Additionally, integrating patient-reported outcomes could provide a more comprehensive view of disease control [57,58].

Ultimately, this model can transform COPD management by shifting from reactive to proactive care, enabling earlier, targeted interventions that optimize resource use and improve patient outcomes. As healthcare systems face growing pressures, such tools offer scalable solutions to enhance patient care and sustainability.

## Figures and Tables

**Table 1:** Regression coefficients of the key variables in the initial model.

	Estimate	Std. Error	z Value	Pr (>|z|)
(Intercept)	−2.2043	0.5714	−3.857	0.000115 ***
SABA+SAMA canisters	0.5733	0.1838	3.119	0.001813 **
Antibiotic courses	4.4836	1.1308	3.965	0.000073 ***

** *p* < 0.01; *** *p* < 0.001; SABA, short-acting β-agonists; SAMA, short-acting muscarinic antagonists.

**Table 2:** Coefficients, bias, and error analysis of the key variables following the LASSO regularization.

	LASSO Corrected Coefficients ^†^	Bias	Std. Error
Constant	−1.873179	2.3224794	0.30133987
SABA+SAMA canisters	0.426672	−0.4253315	0.04596127
Antibiotic courses	3.653875	−3.6681240	0.24263124

^†^ Bootstrap Statistics (R = 1000). SABA, short-acting β-agonists; SAMA, short-acting muscarinic antagonists.

## Data Availability

The original contributions presented in the study are included in the article. Further inquiries can be directed to the corresponding authors.

## References

[1] Global Initiative for Chronic Obstructive Lung Disease (2024). Global Initiative for Chronic Obstrucitve Lung Disease.

[2] Boers E., Barrett M., Su J.G., Benjafield A.V., Sinha S., Kaye L., Zar H.J., Vuong V., Tellez D., Gondalia R. (2023). Global Burden of Chronic Obstructive Pulmonary Disease Through 2050. JAMA Netw. Open.

[3] Buttery S.A.R.A.C., Zysman M.A., Vikjord S.I.A.A., Hopkinson N.I.S., Jenkins C.H., Vanfleteren L.O.E.G.W. (2021). Contemporary Perspectives in COPD: Patient Burden, the Role of Gender and Trajectories of Multimorbidity. Respirology.

[4] Sandelowsky H., Weinreich U.M., Aarli B.B., Sundh J., Høines K., Stratelis G., Løkke A., Janson C., Jensen C., Larsson K. (2021). COPD—Do the Right Thing. BMC Fam Pract..

[5] Stöber A., Lutter J.I., Schwarzkopf L., Kirsch F., Schramm A., Vogelmeier C.F., Leidl R. (2021). Impact of Lung Function and Exacerbations on Health-Related Quality of Life in COPD Patients Within One Year: Real-World Analysis Based on Claims Data. Int. J. Chronic Obstr. Pulm. Dis..

[6] Macintyre N., Huang Y.C. (2008). Acute Exacerbations and Respiratory Failure in Chronic Obstructive Pulmonary Disease. Proc. Am. Thorac. Soc..

[7] Kim V., Aaron S.D. (2018). What Is a COPD Exacerbation? Current Definitions, Pitfalls, Challenges and Opportunities for Improvement. Eur. Respir. J..

[8] Anzueto A. (2010). Impact of Exacerbations on COPD. Eur. Respir. Rev..

[9] Rothnie K.J., Müllerová H., Quint J.K. (2018). Natural History of Chronic Obstructive Pulmonary Disease Exacerbations in a General Practice—Based Population with Chronic Obstructive Pulmonary Disease. Am. J. Respir. Crit. Care Med..

[10] Viniol C., Vogelmeier C.F. (2018). Exacerbations of COPD. Eur. Respir. Rev..

[11] Miravitlles M., Calle M., Molina J., Almagro P., Gómez J.T., Trigueros J.A., Cosío B.G., Casanova C., López-Campos J.L., Riesco J.A. (2022). Spanish COPD Guidelines (GesEPOC) 2021: Updated Pharmacological Treatment of Stable COPD. Arch. Bronconeumol..

[12] Bollmeier S.G., Hartmann A.P. (2020). Management of Chronic Obstructive Pulmonary Disease: A Review Focusing on Exacerbations. Am. J. Health Syst. Pharm..

[13] Zhang J., Chen F., Wang Y., Chen Y. (2023). Early Detection and Prediction of Acute Exacerbation of Chronic Obstructive Pulmonary Disease. Chin. Med. J. Pulm. Crit. Care Med..

[14] Janson C., Wiklund F., Telg G., Stratelis G., Sandelowsky H. (2023). High Use of Short-Acting β2-Agonists in COPD Is Associated with an Increased Risk of Exacerbations and Mortality. ERJ Open Res..

[15] Gondalia R., Bender B.G., Theye B., Stempel D.A. (2019). Higher Short-Acting Beta-Agonist Use Is Associated with Greater COPD Burden. Respir. Med..

[16] Whittaker H., Rubino A., Müllerová H., Morris T., Varghese P., Xu Y., Nigris E.D., Quint J.K. (2022). Frequency and Severity of Exacerbations of COPD Associated with Future Risk of Exacerbations and Mortality: A UK Routine Health Care Data Study. Int. J. Chronic Obstr. Pulm. Dis..

[17] Løkke A., Hilberg O., Lange P., Licht S.D.F., Lykkegaard J. (2022). Disease Trajectories and Impact of One Moderate Exacerbation in Gold B COPD Patients. Int. J. Chronic Obstr. Pulm. Dis..

[18] Løkke A., Hilberg O., Lange P., Ibsen R., Bakke P., Ørts L. (2022). The Impact on Future Risk of One Moderate COPD Exacerbation in GOLD A Patients—A Cohort Study. Eur. Respir. J..

[19] Marott J.L., Çolak Y., Ingebrigtsen T.S., Vestbo J., Nordestgaard B., Lange P. (2022). Exacerbation History, Severity of Dyspnoea and Maintenance Treatment Predicts Risk of Future Exacerbations in Patients with COPD in the General Population. Respir. Med..

[20] Guo J., Chen Y., Zhang W., Tong S., Dong J. (2020). Moderate and Severe Exacerbations Have a Significant Impact on Health-Related Quality of Life, Utility, and Lung Function in Patients with Chronic Obstructive Pulmonary Disease: A Meta-Analysis. Int. J. Surg..

[21] Maltais F., Naya I.P., Vogelmeier C.F., Boucot I.H., Jones P.W., Bjermer L., Tombs L., Compton C., Lipson D.A., Kerwin E.M. (2020). Salbutamol Use in Relation to Maintenance Bronchodilator Efficacy in COPD: A Prospective Subgroup Analysis of the EMAX Trial. Respir. Res..

[22] FitzGerald J.M., Haddon J.M., Bradly-Kennedy C., Kuramoto L., Ford G.T. (2007). Resource Use Study in COPD (RUSIC): A Prospective Study to Quantify the Effects of COPD Exacerbations on Health Care Resource Use among COPD Patients. Can. Respir. J..

[23] Derom E., van Weel C., Liistro G., Buffels J., Schermer T., Lammers E., Wouters E., Decramer M. (2007). Primary Care Spirometry*. Eur. Respir. J..

[24] Chapron A., Lemée T., Pau G., Jouneau S., Kerbrat S., Balusson F., Oger E. (2023). Spirometry Practice by French General Practitioners between 2010 and 2018 in Adults Aged 40 to 75 Years. NPJ Prim. Care Respir. Med..

[25] Navarro Ros F., Maya Viejo J.D. (2024). Preclinical Evaluation of Electronic Health Records (EHRs) to Predict Poor Control of Chronic Respiratory Diseases in Primary Care: A Novel Approach to Focus Our Efforts. J. Clin. Med..

[26] Hurst J.R., Han M.K., Singh B., Sharma S., Kaur G., Nigris E. (2022). De Prognostic Risk Factors for Moderate-to-Severe Exacerbations in Patients with Chronic Obstructive Pulmonary Disease: A Systematic Literature Review. Respir. Res..

[27] Steyerberg E.W. (2019). Clinical Prediction Models.

[28] The R Foundation for Statistical Computing The R Project for Statistical Computing. https://www.r-project.org/.

[29] Schneeweiss S., Avorn J. (2005). A Review of Uses of Health Care Utilization Databases for Epidemiologic Research on Therapeutics. J. Clin. Epidemiol..

[30] Ranganathan P. (2021). An Introduction to Statistics: Choosing the Correct Statistical Test. Indian J. Crit. Care Med..

[31] Tibshirani R. (1996). Regression Shrinkage and Selection via the Lasso. J. R. Stat. Soc..

[32] Chicco D., Jurman G. (2020). The Advantages of the Matthews Correlation Coefficient (MCC) over F1 Score and Accuracy in Binary Classification Evaluation. BMC Genom..

[33] Soler-Cataluña J.J., Izquierdo J.L., Juárez Campo M., Sicras-Mainar A., Nuevo J. (2023). Impact of COPD Exacerbations and Burden of Disease in Spain: AVOIDEX Study. Int. J. Chronic Obstr. Pulm. Dis..

[34] Gönen M. (2004). Sample Size and Power for McNemar’s Test with Clustered Data. Stat. Med..

[35] Lachenbruch P.A. (1992). On the Sample Size for Studies Based upon McNemar’s Test. Stat. Med..

[36] Franssen F.M.E., Alter P., Bar N., Benedikter B.J., Iurato S., Maier D., Maxheim M., Roessler F.K., Spruit M.A., Vogelmeier C.F. (2019). Personalized Medicine for Patients with COPD: Where Are We?. Int. J. COPD.

[37] López-Campos J.L., Hartl S., Pozo-Rodriguez F., Roberts C.M. (2013). European COPD Audit: Design, Organisation of Work and Methodology. Eur. Respir. J..

[38] Perret J., Yip S.W.S., Idrose N.S., Hancock K., Abramson M.J., Dharmage S.C., Walters E.H., Waidyatillake N. (2023). Undiagnosed and “overdiagnosed” COPD Using Postbronchodilator Spirometry in Primary Healthcare Settings: A Systematic Review and Meta-Analysis. BMJ Open Respir. Res..

[39] Wedzicha J.A., Seemungal T.A.R. (2007). COPD Exacerbations: Defining Their Cause and Prevention. Lancet.

[40] Donaldson G.C., Wedzicha J.A. (2006). COPD Exacerbations.1: Epidemiology. Thorax.

[41] Hatipoglu U.S., Aboussouan L.S. (2016). Treating and Preventing Acute Exacerbations of COPD. Clevel. Clin. J. Med..

[42] David B., Bafadhel M., Koenderman L., De Soyza A. (2021). Eosinophilic Inflammation in COPD: From an Inflammatory Marker to a Treatable Trait. Thorax.

[43] Archontakis Barakakis P., Tran T., You J.Y., Hernandez Romero G.J., Gidwani V., Martinez F.J., Fortis S. (2023). High versus Medium Dose of Inhaled Corticosteroid in Chronic Obstructive Lung Disease: A Systematic Review and Meta-Analysis. Int. J. Chronic Obstr. Pulm. Dis..

[44] Stolbrink M., Bonnett L.J., Blakey J.D. (2019). Antibiotics for COPD Exacerbations: Does Drug or Duration Matter? A Primary Care Database Analysis. BMJ Open Respir. Res..

[45] Fan V.S., Gylys-colwell I., Locke E., Sumino K., Nguyen H.Q., Thomas R.M., Magzamen S. (2016). Overuse of Short-Acting Beta-Agonist Bronchodilators in COPD during Periods of Clinical Stability. Respir. Med..

[46] Singh H., Schiff G.D., Graber M.L., Onakpoya I., Thompson M.J. (2017). The Global Burden of Diagnostic Errors in Primary Care. BMJ Qual. Saf..

[47] Sharafkhaneh A., Altan A.E., Colice G.L., Hanania N.A., Donohue J.F., Kurlander J.L., Rodriguez-Roisin R., Altman P.R. (2014). A Simple Rule to Identify Patients with Chronic Obstructive Pulmonary Disease Who May Need Treatment Reevaluation. Respir. Med..

[48] Negewo N.A., Gibson P.G., Wark P.A., Simpson J.L., McDonald V.M. (2017). Treatment Burden, Clinical Outcomes, and Comorbidities in COPD: An Examination of the Utility of Medication Regimen Complexity Index in COPD. Int. J. Chronic Obstr. Pulm. Dis..

[49] Shen N., He B. (2014). Personalized Medicine in COPD Treatment. Curr. Respir. Care Rep..

[50] Wouters E.F.M., Wouters B.B.R.A.F., Augustin I.M.L., Franssen F.M.E. (2017). Personalized Medicine and Chronic Obstructive Pulmonary Disease. Curr. Opin. Pulm. Med..

[51] Alí A., Giraldo-Cadavid L.F., Karpf E., Quintero L.A., Aguirre C.E., Rincón E., Vejarano A.I., Perlaza I., Torres-Duque C.A., Casas A. (2019). Frequency of Emergency Department Visits and Hospitalizations Due to Chronic Obstructive Pulmonary Disease Exacerbations in Patients Included in Two Models of Care. Biomedica.

[52] Kirenga B.J., Alupo P., van Gemert F., Jones R. (2023). Implication of the Global Initiative for Chronic Obstructive Lung Disease 2023 Report for Resource-Limited Settings: Tracing the G in the GOLD. Eur. Respir. J..

[53] Bailey K.L. (2012). The Importance of the Assessment of Pulmonary Function in COPD. Med. Clin. N. Am..

[54] Vallée A. (2023). Digital Twin for Healthcare Systems. Front. Digit. Health.

[55] Khalifa M., Albadawy M. (2024). Artificial Intelligence for Clinical Prediction: Exploring Key Domains and Essential Functions. Comput. Methods Programs Biomed. Update.

[56] Krishnan G., Singh S., Pathania M., Gosavi S., Abhishek S., Parchani A., Dhar M. (2023). Artificial Intelligence in Clinical Medicine: Catalyzing a Sustainable Global Healthcare Paradigm. Front. Artif. Intell..

[57] Quittner A.L., Nicolais C.J., Saez-Flores E., Wilmott R., Bush A., Deterding R., Ratjen F., Sly P., Zar H., Li A.P. (2019). Integrating Patient-Reported Outcomes Into Research and Clinical Practice. Kendig’s Disorders of the Respiratory Tract in Children.

[58] Siu D.C.H., Gafni-Lachter L. (2024). Addressing Barriers to Chronic Obstructive Pulmonary Disease (COPD) Care: Three Innovative Evidence-Based Approaches: A Review. Int. J. Chronic Obstr. Pulm. Dis..

